# A New Path-Constrained Rendezvous Planning Approach for Large-Scale Event-Driven Wireless Sensor Networks

**DOI:** 10.3390/s18051434

**Published:** 2018-05-04

**Authors:** Ahmadreza Vajdi, Gongxuan Zhang, Junlong Zhou, Tongquan Wei, Yongli Wang, Tianshu Wang

**Affiliations:** 1 School of Computer Science and Engineering, Nanjing University of Science and Technology, Nanjing 210094, China; vajdi@njust.edu.cn (A.V.); gongxuan@njust.edu.cn (G.Z.); yongliwang@njust.edu.cn (Y.W.); wangtianshu122@163.com (T.W.); 2 Department of Computer Science and Technology, East China Normal University, Shanghai 200062, China; tqwei@cs.ecnu.edu.cn

**Keywords:** mobile sink, energy efficiency, fuzzy decision system, wireless sensor network, rendezvous point planning

## Abstract

We study the problem of employing a mobile-sink into a large-scale Event-Driven Wireless Sensor Networks (EWSNs) for the purpose of data harvesting from sensor-nodes. Generally, this employment improves the main weakness of WSNs that is about energy-consumption in battery-driven sensor-nodes. The main motivation of our work is to address challenges which are related to a network’s topology by adopting a mobile-sink that moves in a predefined trajectory in the environment. Since, in this fashion, it is not possible to gather data from sensor-nodes individually, we adopt the approach of defining some of the sensor-nodes as Rendezvous Points (RPs) in the network. We argue that RP-planning in this case is a tradeoff between minimizing the number of RPs while decreasing the number of hops for a sensor-node that needs data transformation to the related RP which leads to minimizing average energy consumption in the network. We address the problem by formulating the challenges and expectations as a Mixed Integer Linear Programming (MILP). Henceforth, by proving the NP-hardness of the problem, we propose three effective and distributed heuristics for RP-planning, identifying sojourn locations, and constructing routing trees. Finally, experimental results prove the effectiveness of our approach.

## 1. Introduction

**Figure 1:**
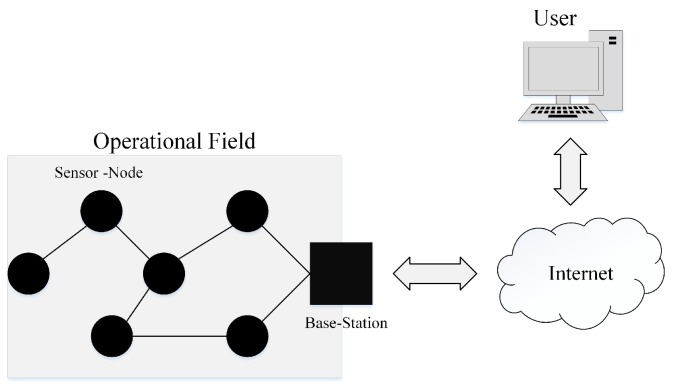
Application of WSNs in IoT.

Advances in electronic industry and producing sensors with high sensing and processing abilities could bring promising applications to various aspects of today’s life; mainly, high-tech industries, healthcare systems, military, agriculture, and so on [1,2]. Sensor-nodes are able to communicate with each other or with other devices to share their sensing information. Therefore, diverse types of network topologies can be constructed in the category of Wireless Sensor Networks (WSNs) [3]. Due to very vast developments in the Internet technology around the world, people become more interested to bring the Internet to all of the aspects of life. Indeed, there is no limitation for the applications that the Internet can involve for changing the style of humans’ life. This very big goal could become achievable by the idea of IoT (Internet of Things) which was proposed in 1999 by Kevin Ashton [4,5]. As shown in Figure 1, WSNs play a major role in IoT since sensor-nodes can provide the opportunity to observe and monitor any environment. Due to limited capabilities of the sensor-nodes and complexity of the environments; in most of the applications, a standalone sensor-node cannot connect to the Internet directly. Therefore, a WSN can work as a bridge between the sensor-nodes and the Internet. Clearly, any improvement in WSNs can extend the usability of IoT especially new improvements in large-scale WSNs which is our main concern in the current work [6].

In large scale WSNs, hundreds or even thousands of sensor-nodes are being distributed in harsh and unattended environments to capture natural phenomena and their changes. Due to the use of mostly non-rechargeable batteries in the sensor-nodes, balancing energy consumption is the main design issue that can guarantee the lifetime and reliability of the network. One of the common problems that is related to nonuniform energy depletion is hot-spot phenomenon in that the sensor-nodes which are closer to the base-station will die earlier in contrast to the sensor-nodes which are located far from the base-station by maintaining more than 90% of their initial energy [7].

Recently, employing mobile-sink(s) in the network becomes one of the efficient solutions to balance energy-consumption among the sensor-nodes [8,9,10,11,12,13]. The main idea behind this solution is that the mobile-sink(s) can move across the network and harvest the data from the sensor-nodes and reduce or eliminate the number of hops in the fashion of multi-hop communication. Beside the simplicity of this idea, due to the limitations of mobile-sink as a vehicle that should move in a physical environment and the scale of WSNs, there are two main challenges which are in the state of research. The first challenge is about the fashion of movement for the mobile-sink(s) in the network. And the second one addresses the visiting strategy of the mobile-sink(s) that should visit all of the sensor-nodes one-by-one or adopting a visiting approach for a set of sensor-nodes which work as Rendezvous Points (RPs) in the network. Indeed, the application scenario is the most important factor that can lead to different considerations.

Due to enough processing capabilities in most of the sensor-nodes recently, Event-Driven Wireless Sensor Networks (EWSNs) are introduced in [14,15]. Compare to typical WSNs that each sensor-node is responsible for capturing measurement values and sending them to higher layers for further processing; In EWSNs, all of the sensor-nodes are able to process the measurement values individually, and then by recognizing an event, report the event-alarm to higher layers. Based on the nature of events in the environment, the sensor-nodes should be equipped with different algorithms for event detection. For example, some events can be defined based observing a value more than a predefined threshold such as (temperature ≥ 70 °C). Therefore, a sensor-node triggers an event alarm by capturing a value more than the threshold. Some events can be more complicated which require some advance detection techniques like supervised or unsupervised techniques from machine learning that is not in scope of our current research. In our approach, we focus on delivering the event-alarms to the system’s operators. There are two main advantages for EWSNs in contrast to the typical WSNs; first, in WSNs for recognizing an event all of the information should be transferred to a central processing station and then due to the size of data-set by applying advanced analyzing methods the event information can be extracted which is expensive in time and computational resources. The second advantage is about extending the lifetime of the network by reducing unnecessary communications.

Herein, by considering EWSNs for the purpose of monitoring a large operational field such as forest and detecting some specific events such as fire, we employ a grounded mobile-sink for data harvesting. Obviously, the mobile-sink cannot move in an arbitrary trajectory due to physical obstacles. Therefore, we assume that there is a predefined trajectory in the environment. In addition, based on the development scale, practically it is not possible to install the sensor-nodes manually. Thus, we employ a random deployment fashion such as by aircraft. By considering the practical constraints of the environment and the mobile-sink, there is not any possibility for the mobile-sink to visit all of the sensor-nodes one-by-one. Accordingly, the sensor-nodes which are located in proximity of the mobile-sink’s trajectory can work as RPs in the network.

Based on the above mentioned statements about the intended application scenarios, our main objective is to select a set of RPs in periphery of the predefined trajectory that can satisfy two main expectations which are minimizing energy-consumption of the sensor-nodes and reducing the average data delivery delay in the network. Distributed nature of WSNs and inability of the system to perform any centralized solution is another important consideration in our proposed approach. Therefore, we could achieve the following contributions in our work:

- Modeling the problem by a Mixed Integer Linear Programming (MILP).
- Analyzing the theoretical aspects of the problem and proving its NP-hardness.
- Proposing distributed and comprehensive heuristics for constructing a comprehensive framework in our approach.
- The first heuristic determines a suitable set of RPs. To cope with the uncertainties in the environment, we designed a Fuzzy Decision System (FDS) model for identifying the potentiality of a sensor-node for becoming a RP.
- After selecting the RPs, our second heuristic attempts to find appropriate locations for the mobile-sink to stop and collect the data from the sensor-nodes which are called “sojourn locations”.
- And the third heuristic constructs routing trees for the sensor-nodes which cannot contact with the mobile-sink directly. These sensor-nodes transfer their data to the related RP.
- Our experimental results prove the effectiveness of our approach.

The rest of the paper is organized as follows. Section 2 discusses about some of the prominent related works. Section 3 summarizes the essential preliminaries for our work. Section 4 introduces the problem and its constraints in the formal form. Section 5 proposes our three heuristics to construct a comprehensive framework. Section 6 presents the numerical experiments. And finally Section 7 concludes this paper.

## 2. Related Work

Based on a study in [16], data communication for a sensor-node with other entities in the network consumes a large amount of energy in contrast with data processing inside the sensor-node. This axiom is the main lead to motivate for designing routing protocols in WSNs. As one of the significant works that could influence further researches in the area, in [17,18], Heizenlman et al. proposed an approach to select some of the sensor-nodes as cluster-heads (CHs) and dismiss other non-CHs from communicating with the base-station directly that is called LEACH (Low-Energy Adaptive Clustering Hierarchy). Therefore, the sensor-nodes which are not selected as CHs should be allocated to a suitable CH. In LEACH, by defining the concept of round and implementing a probabilistic technique in each round, a set of sensor-nodes will be selected as CHs. In the next round, a new set of CHs will be selected. And after determining the set of CHs, other sensor-nodes will join to the closest CHs by considering signal strength. Despite the improvement of the approach in saving energy, the main disadvantage is its probabilistic nature without considering the location of the CHs and some other parameters such as residual energy. Therefore, it is probable that some CHs locate in the margin of the cluster and being far from the base-station. In addition, LEACH assumes that all of the sensor-nodes can contact with the base-station directly which is impractical for large-scale WSNs. To improve the accuracy of cluster-head selection by injecting some related information to the process of selection, scholars implemented different routing protocols based on probabilistic models [19,20], swarm intelligence [21,22,23], and fuzzy systems [24,25,26]. However, in the mentioned efforts the main assumption is all of the sensor-nodes and the base-station are stationary after installation. Recently, with improvement in the new technologies, the idea of utilization a(some) mobile-sink(s) in the network is proposed in [27,28] to harvest data from the sensor-nodes in the environment.

Application of mobile-sink can improve lifetime and reliability of the network by controlling energy depletion in a uniform manner which can prevent energy holes [9,29]. The main research question that arises is *“the fashion of data-harvesting which the mobile-sink(s) should follow”*. Solving this question essences to divide the question to two sub-questions. The first sub-question should determine the *“visiting strategy”*. And the second issue refers to the *“movement strategy”*. The visiting strategy can be divided to “Direct” and “Indirect”. And the movement strategy can be considered as “Constrained” and “Unconstrained”. In the following, we will review some of the prominent works for each division.

In direct approaches, the main strategy is to visit all of the sensor-nodes one-by-one. By adopting this manner, the movement strategy for the mobile-sink in case of large-scale WSNs should be unconstrained. Since in case of constrained movement, the sensor-nodes should be installed manually in the periphery of a predefined trajectory in which the mobile-sink can reach all of the sensor-nodes one-by-one. As one of the early efforts, in [30], Shah et al. defined “data mules” such as people, animals, or vehicles in the environment which are equipped with related devices for data-harvesting. Since the main purpose of existence of data mules in the environment is not working as mobile-sink, the random walk model is adopted to model their movement fashion. Obviously, the nature of random walk can’t guarantee that all of the sensor-nodes can be visited by data mules. In [31], Ma et al. proposed another solution to visit the sensor-nodes directly. Their approach also can be extended to multiple mobile-sink(s). They named the problem as the single-hop data gathering problem (SHDGP). For each tour distance/time is being considered as the constraint for the mobile-sink(s) and then by formulating the SHDGP problem into a mixed integer program its NP-hardness is proved. Afterward, a tour-planning heuristic is proposed. The authors claimed that their solution is suitable for large-scale networks, however, it is not practical to visit a large number of sensor-nodes one-by-one with considering distance/time constraint.

To overcome the problem of direct approaches for large-scale WSNs, the idea of defining a set of sensor-nodes as Rendezvous Points (RPs) is proposed and still it is under development by considering different assumptions and employing new technologies in [9,11,32,33,34]. Generally, the mobile-sink visits the RPs instead of visiting the sensor-nodes one-by-one and other sensor-nodes will be assigned to suitable RPs that can minimize their communication range. In [32], Xing et al. developed two rendezvous planning algorithm which are RP-CP and RP-UG. RP-CP attempts to determine the moving trajectory of the mobile-sink and RPs by constructing the data routing tree in the base-station. Then, the algorithm starts to find the most important edges in the tree. The importance of an edge is measured by the number of source-to-root paths from the edge. The mobile-sink starts its tour by passing from the most important edges and other sensor-nodes which are not located on the tour, transfer their data to the closest RP which is located on the tour. In this approach, the movement strategy is limited to the routing tree. However, in RP-UG the main motivation is to assume that the mobile-sink can move freely in the network. RP-UG is a utility-based greedy heuristic which improves itself by iterations. In each new iteration, the previous tour will be extended by adding new RPs to the tour until satisfying the maximum tour length. And the utility is defined as the trade-off between the mount of energy that can be saved by adding a RP and the length of the tour that is being increased. To discuss about some of the disadvantages of RP-CP and RP-UG, in RP-CP constructing the data routing tree in the base-station requires for obtaining the position of sensor-nodes which is an expensive (by considering time and energy) process in case of large-scale WSNs. And for RP-UG two practical scenarios might happen. In the first one, the base-station gathers the position of sensor-nodes and runs the algorithm which is not practical for large-scale networks. And in the second scenario the mobile-sink starts to move in the environment to determine the routes and selects the RPs which will be a time-consuming process.

In [35], Chatzigiannakis et al. studied the problem of mobility in wireless sensor networks as one of the early efforts by proposing different patterns along with data collection strategies. Their extensive numerical simulations could prove the effectiveness of employing mobile-sink in the network. In addition, in [36], Chatzigiannakis et al. investigated the problem of applying multiple mobile-sinks in the network by identifying its characteristics. They proposed three protocols based on different degrees of network knowledge. And in [37], Chatzigiannakis et al. provided a toolkit to simulate the application of mobile-sink in WSNs.

In [9], Salarian et al. focused on determining the RPs and the trajectory for the mobile-sink in a distributed fashion. In the approach, the sensor-nodes with highest weight will be selected as RPs that can reduce the number of multi-hop communications in the network and conserve energy. The weight of each sensor-node will be obtained by its workload. And finally by applying the classic TSP solver, the trajectory for the mobile-sink is being calculated in the approach by considering a threshold value for tour length to guarantee data delivery delay time. In [38], Yang et al. consider the problem of defining suitable trajectories for the mobile-sink by assuming the random distribution of the sensor-nodes. In their approach, the network is divided to subareas and based on the node density the trajectory for the mobile-sink will be constructed by following the Hilbert space-filling curve. And at last, the trajectories in the subareas will be combined together for acquiring the whole network’s trajectory. In [34], Qadori et al. studied the problem of implementing multiple mobile-sinks in the network based on the agent spawning by defining a main mobile-sink that is able to swam other mobile-sinks with various task assignments.

In [39], Konstantopoulos et al. concerned the problem of defining a fix trajectory for the mobile-sink in an urban area by mounting the sink-node on the public bus. For transferring data from any arbitrary sensor-node in the network to the mobile-sink, a hierarchical structure is proposed by defining some of the sensor-nodes as cluster-heads (CHs) and another type of sensor-nodes which are called Rendezvous Nodes (RNs). The CHs are responsible to harvest data from the sensor-nodes and directly or by multi-hop communications transfer it to the related RN. The RNs work as bridge and buffer the data until the mobile sink reaches to the communication-range of the RN. The selection of CHs is based on the technique that is propose in [40] by Chen et al. The process of identifying suitable RNs will be conducted in each cluster that its cluster-head can contact with the mobile-sink directly. Generally, specifying a RN for a cluster is based on weight-assignment for each candidate sensor-nodes by considering two parameters which are the receipt time of the first and last BEACON message. Since the process for locating the candidate sensor-nodes is random, it cannot guarantee to find optimal solution based on the position of the sensor-nodes in the network. In addition, among the candidate sensor-nodes, the final CHs will be selected based on the highest residual energy without considering other parameters such as location or the number of neighbor sensor-nodes.

In [11], Tang et al. designed a solution by setting the maximum number of multi-hop communications in the network as the main measure to design CHs in the network. Obviously, when the number of hops is zero then the problem becomes visiting all of the sensor-nodes one-by-one and can be solved by traditional approaches such as TSP. By increasing the number of multi-hop communications, the main idea is to solve the problem for smaller parameters and extend the solution for larger parameters. The nature of the proposed approach requires processing unit which makes it unadaptable for large-scale networks in which it is not possible to obtain global information about the location and status of the sensor-nodes.

In [41,42], the authors attempt to consider the concept of event for developing mobile sinks in the network. In [41], Tashtarian et al. proposed a convex mathematical model inspired by the support vector regression (SVR) technique to design a COT (Continuous and Optimal Trajectory) that considers three main factors which are the group of sensor-nodes that capture events, the velocity of the mobile sink, and reporting time slot. They defined their model without considering any predefined structure for the network. In [42], Ota et al. worked on another approach which attempts to control the movement of mobile sink based on event prediction. It predicts an event from collected sensory data by utilizing the maximum likelihood estimation. And then it designs the movement policy of the mobile sink based on reinforcement learning in Markov decision process. Event prediction is promising approach to improve reliability and life time of the network; however the proposed approach assumes spatial correlation among sensor nodes. Therefore, sensory data are being described by normal distribution. Generally, prediction based on spatial correlation has two disadvantages: firstly, it limits the prediction range to local areas which means if an event occurs far from the mobile sink in large scale networks the approach will not be able to predict it. Secondary, it is not applicable for all types of events. For example, some events just happen in one point of the network or their expansion rate is so slow and neighbor sensor-nodes may not capture in time. Thus, because of relying on spatial correlation for event prediction the proposed approach is weak in these scenarios.

**Figure 2:**
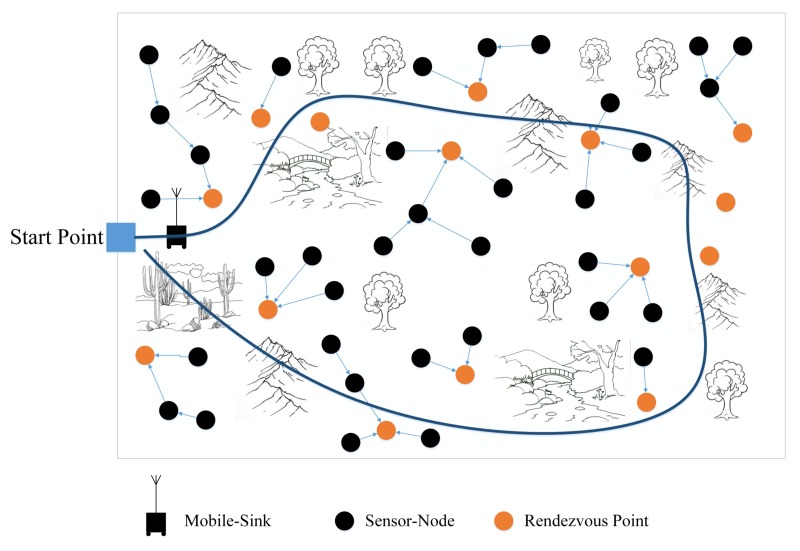
An application case for EWSNs with Mobile-Sink.

In our work, we consider an application case that conforms to the real conditions in the environment. As an example which is illustrated in Figure 2, in some operational field, the grounded mobile-sink cannot move in an arbitrary trajectory. Obviously by this constrain, the only solution that can minimize data delivery time is to reduce the number of stop-points (sojourn locations) in the trajectory for the mobile-sink. However, reducing the number of sojourn locations will lead the sensor-nodes to use multi-hop communication manner that can increase energy consumption in the network. Our goal is to find a balanced solution between the number of RPs, sojourn locations, and energy-consumption in the network which will be explained with details in the next sections.

## 3. Preliminaries

In this section, we focus on illustrating the general communication and energy consumption models in the network along with identifying the basic assumptions that can lead to our approach for finding the most optimal solution.

### 3.1. Network Model

In our model, a large number of sensor-nodes are being distributed in a large target field which are represented by $S=\left\{s_{1},\ldots ,s_{n}\right\}$ with the ability to recognize the events individually. By considering the large-scale of the environment, it is not practical to install the sensor-nodes manually. Therefore, we adopt a random distribution manner such as by aircraft. To conserve energy and for improving the network’s security, we assume that the sensor-nodes do not contact with any global positioning system to obtain their accurate locations in the network. Instead, they just contact with their neighbor sensor-nodes which are located in their communication range and keep the position by $PS=\left\{ps_{i}\left(\theta_{i},d_{i}\right)|\forall i=1,\ldots ,n\right\}$ where θ represents the direction of neighbor sensor-node and *d* indicates its Euclidean distance. In addition, the following assumptions are being considered in our model [43,44,45,46,47]:

1. A target field without limitation based on geographical differences.
2. One mobile-sink in the network.
3. The sensor-nodes have enough computational capabilities and are able to run our proposed solution.
4. Each sensor-node has a unique ID which is known for the mobile-sink and the base-station.
5. The sensor-nodes and the base-station are stationary after installation in the target field.
6. The network is homogenous that means all of the sensor-nodes are in the same type. And in the beginning of network’s operation, they have the same amount of initial energy.
7. We assume that the conditions of MAC layer are ideal and communication links between sensor-nodes can establish or cannot. In case of establishment, the sensor-nodes can contact with each other with high quality (based on the standards for wireless communication) (This is an ideal assumption which might not be practical in real wireless communication fashion. The reason for this assumption is to facilitate our simulation. Generally, if two sensor-nodes or a sensor-node with the mobile-sink cannot contact with each other based on the expected quality, they will not be considered in the communication range of each other).
8. The sensor-nodes and the mobile-sink are able to control their transmission power to the desired destination (This assumption is arguable in practical implementations. However, the correctness of our assumption just implies that the sensor-nodes can conserve their energy by adjusting their transmission power and operate longer in the network. Our proposed approach does not rely on this assumption).
9. The sensor-nodes and the mobile-sink are able to calculate the distance from the received signal strength (Some scholars might doubt on the possibility of this assumption. Indeed, our approach does not need the accurate distance between the sensor-nodes. It is just important to recognize the neighbor sensor-nodes, correctly).
10. The radio link is symmetric which means energy consumption for data transmission from node A to node B is same as from node B to node A (This assumption is ideal. We relied on this assumption because of facilitating our simulation).
11. We neglect communication time between the sensor-nodes.

### 3.2. Energy Model

Wireless channel model is another important factor that should be formulated to identify energy consumption for communication between the sensor-nodes or the sensor-nodes with the mobile-sink. In our research, we adopt the first order radio model in [48]. The model defines a threshold value, Equation (3) which establishes two different fashions for the communication model. If the distance between transmitter and receiver is less than $d_{0}$, then we adopt the free space model ($d^{2}$ power loss). Otherwise the multipath fading channel is being considered ($d^{4}$ power loss) as shown in the following equations.

(1) $$E_{Tx}=\left(l,d\right)=\left\{\begin{array}{cc} l\times E_{elec}^{Tx}+l\times \xi_{fs}\times d^{2} & d<d_{0}\\ l\times E_{elec}^{Tx}+l\times \xi_{fs}\times d^{4} & d\geq d_{0} \end{array}\right.$$

(2) $$E_{Rx}\left(l\right)=l\times E_{elec}^{Rx}$$

where $E_{elec}^{Tx}$ and $E_{elec}^{Rx}$ are the energy consumption per bit in the transmitter and receiver circuits. *l* refers to bit transformation for the sensor-node. In addition, $\xi_{fs}$ and $\xi_{fmp}$ are the energy consumption factor of amplification for the free space and multipath radio models, respectively. The threshold value $d_{0}$ is defined as follows.

(3) $$d_{0}=\sqrt{\frac{\xi_{fs}}{\xi_{mp}}}.$$

## 4. Problem Statement and Formulation

In this section, we state the formal representation of our problem. And then by a Mixed Integer Linear Programming (MILP) formulation the NP-hardness of the problem will be proved. We also discuss about limitations and constrains that the final solution should follow.

### 4.1. Problem Definition

By considering an arbitrary trajectory Γ for the mobile-sink which is predefined in the environment, our problem can be divided to three sub-problems for constructing a comprehensive framework. The first and the second sub-problems which are RP-planning and identifying sojourn locations are related together and cannot be considered independently (We argue that by our assumption (fixed trajectory) it is not possible to determine the sojourn locations before determining the rendezvous points. Because in case of irrelevant selection for some of the sojourn locations, two situations might happen. In the first situation, the rendezvous points should transfer their message to the irrelevant sojourn locations with high energy consumption. Furthermore, in the second situation, some of the rendezvous points cannot reach to any sojourn location which will make the network’s operation impractical). And the third sub-problem is about constructing routing trees to transfer data from the rest of sensor-nodes to the related RP.

Planning a set of RPs $\Delta =\left\{\delta_{1},\ldots ,\delta_{m}\right\}$ such that m≤|S| in the proximity of Γ is our first task. Then our problem becomes identifying sojourn locations Ψ for the mobile-sink to stop and harvest data from RPs. However, due to continuous nature of the trajectory Γ→∞, we have to find a finite set of sojourn locations $\Psi =\left\{\psi_{1},\ldots ,\psi_{z}\right\}$ such that 1≤z≤|S|. And as it is describe in the following expression, the RPs should be assigned to an appropriate sojourn location.

(4) Δ→Ψ,

Such that:

(5) $$\forall i=1,\ldots ,m;\;\forall j=1,\ldots ,z;\;\forall k=1,\ldots ,z;\;m\geq z\;\mathrm{and}\;m,z\leq |S|\;if\;\delta_{i}\to \psi_{j}\wedge \delta_{i}\to \psi_{k}\Rightarrow j=k.$$

And finally, connecting the rest of sensor-nodes to appropriate RPs is the final step which will be addressed in the current work.

(6) S−Δ→Δ.

### 4.2. MILP Formulation

In this section, we describe our Mixed Integer Linear Programming (MILP) formulation with identifying the objective of the problem and the related constrains.

#### 4.2.1. Objective

The main objective of our problem’s formulation is to minimize Data Delivery Time (DDT) for each sensor-node as illustrated in the following.

(7) $$DDT\left(i\right)=N_{T}\left(i,j\right)+W_{T}\left(i,j\right),$$

(8) $$N_{T}\left(i,j\right)=N_{h}\left(i,j\right)\times T_{h}.$$

where i=1,…,n and j=1,…,m. $N_{T}$ stands for the required time for the sensor-node *i* to deliver its data to the related RP *j* based on number of hops that can be obtained by Equation (9) in which $N_{h}$ represents the number of hops and $T_{h}$ is the required time for each hop to process the related information and transfer the data to the next sensor-node. We consider a constant value in $T_{h}$ for the sensor-nodes since this value is small and roughly same for all of the sensor-nodes. And at last $W_{T}$ indicates the waiting time for the buffered data which is being sent by the sensor-node *i* to the RP *j* for delivering to the mobile-sink.

Compared to the approaches which assume a constant velocity for the mobile-sink to move in the environment [41], we suppose a flexible velocity for the mobile-sink. More precisely, between two consecutive sojourn locations, the mobile-sink can move faster and decrease waiting time or physical obstacles can increase waiting time. We believe considering a constant velocity or predictable velocity for the mobile-sink is suitable for typical WSNs for the purpose of continuous environmental monitoring. Because the sensor-nodes can be informed about the time that the mobile-sink arrives to the sojourn location and adjust data transformation time. However, in EWSNs there is not any pre-information that when an event may occur. Therefore, the movement of mobile-sink might not follow a pre-determined manner and due to some emergency happenings the movement fashion should be changed to decrease the waiting time for improving reliability of the network.

In addition, the number of sojourn locations has direct influence on reliability and energy- consumption in the network which is hidden in our objective function. Obviously, the ideal scenario for decreasing the waiting time is to specify just one sojourn location in the network. However, this assumption will increase the energy-consumption by communication overhead among the sensor-nodes. Extremely increasing the number of sojourn locations will decrease energy-consumption but it will increase the waiting time. Therefore, our main objective is to find a balanced combination between energy-consumption and reliability in the network by minimizing the data delivery time in Equation (7).

#### 4.2.2. Constraints

**Figure 3:**
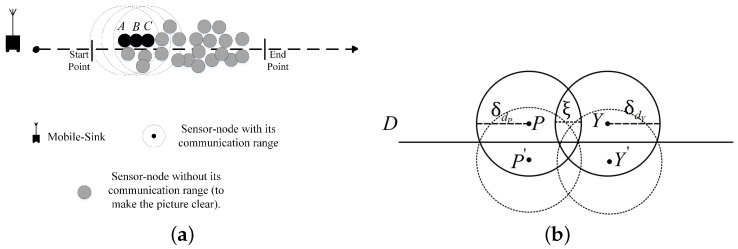
(**a**) An example of dense distribution of the sensor-nodes in the periphery of the trajectory. (**b**) Two consecutive sensor-nodes that work as RP and their counterparts in other side of the trajectory.

To guarantee proper selection of sojourn locations and RPs and assigning the rest of sensor-nodes to sufficient RPs that can minimize the data delivery time, the following constrains must be met:

1. *The maximum number of RPs on the trajectory should not exceed $P_{max}$*.
  (9) $$\left|\Delta \right|\leq P_{max},$$
  **(Note) The method for calculating the maximum number of RPs:**
  Since the trajectory of the mobile-sink is pre-defined to obtain the maximum number of RPs, we assume in an ideal case that all of the RPs are located beside the trajectory with dense distribution as illustrated in Figure 3a in which, as an example, the sensor-nodes *A*, *B* and *C* are in the communication range of each other and all of them can contact with the mobile-sink directly. However, among these three sensor-nodes one of them should contact with the mobile-sink as RP and other two sensor-nodes should transfer their data to the selected RP. Otherwise, the mobile-sink should collect the data from all of RPs in the periphery of the trajectory that consumes a considerable amount of time in every tour. Thus, the maximum number of RPs can be obtained as follows.
  (10) $$P_{max}=\left(\frac{D}{(2\times \delta_{d_{P}}+2\times \delta_{d_{Y}})-\xi }\times 2\right)\times 2.$$
  such that $\delta_{d_{P}}$ and $\delta_{d_{Y}}$ are two consecutive sensor-nodes as depicted in Figure 3b,
  In Equation (10), *D* represents the total distance of the trajectory. By assuming dense distribution of the sensor-nodes in the periphery of the trajectory, the maximum number of RPs can be obtained by dividing the total distance *D* to the summation of communication diameter of the two consecutive sensor-nodes with subtracting from the intersected line that is represented by ξ. The obtained value should be multiplied by 2 because in this part of the trajectory two RPs should be selected. And by considering the symmetry condition in another side of the trajectory, the obtained value should be multiplied by 2. The value of ξ is user-defined. Obviously, due to assuming dense distribution of the sensor-nodes in the whole trajectory, we need to consider two arbitrary consecutive sensor-nodes and the obtained value will be same for rest of the sensor-nodes.
2. *To optimize energy-consumption in communication between sensor-nodes, the solution should not contain energy-triangles.* For example, if sensor-node *A* can contact with sensor-node *C* directly, then sensor- node *B* cannot play hop-role between them because $E_{AC}\leq E_{AB}+E_{BC}$. The following expressions illustrate the constraint formally.
  ∀i=1,…,n, ∀j=1,…,n and i≠j:
  (11) $$F_{i}\left(s_{j}\right)=\left\{\begin{array}{cc} 1 & \mathrm{if}\mathrm{sensor}\;j\;\mathrm{is}\mathrm{in}\mathrm{the}\mathrm{communication}\;\mathrm{range}\mathrm{of}\mathrm{sensor}\;i,\\ 0 & \mathrm{else}. \end{array}\right.$$
  (12) $$H_{i}\left(s_{j}\right)=\left\{\begin{array}{cc} 1 & \mathrm{if}\mathrm{sensor}\;i\;\mathrm{contacts}\mathrm{with}\mathrm{sensor}\;j\;\mathrm{directly},\\ 0 & \mathrm{else}. \end{array}\right.$$
  (13) $$F_{i}\left(s_{j}\right)\times H_{i}\left(s_{j}\right)=1.$$
3. The selected RP should be in communication range of the sojourn location.
  ∀i=1,…,z and ∀j=1,…,m:
  (14) $$J_{i}\left(s_{j}\right)=\left\{\begin{array}{cc} 1 & \mathrm{if}\mathrm{sensor}\;j\;\mathrm{is}\mathrm{in}\mathrm{the}\mathrm{communication}\;\mathrm{range}\mathrm{of}\mathrm{sojourn}\mathrm{location}\;i,\\ 0 & \mathrm{else}. \end{array}\right.$$
  (15) $$\Pi_{i}=\left\{\begin{array}{cc} 1 & \mathrm{if}\mathrm{sensor}\;j\;\mathrm{is}\mathrm{RP},\\ 0 & \mathrm{else}. \end{array}\right.$$
  (16) $$J_{i}\left(s_{j}\right)\times \Pi_{i}=1.$$
4. Each sensor-node should send its data to only one RP for preventing data redundancy and extra energy-consumption in the network.
  ∀i=1,…,n and ∀j=1,…,m:
  (17) $$A_{j}\left(s_{i}\right)=\left\{\begin{array}{cc} 1 & \mathrm{if}\;\mathrm{sensor}\;i\;\mathrm{is}\;\mathrm{assigned}\;\mathrm{to}\;\mathrm{RP}\;j,\\ 0 & \mathrm{else}. \end{array}\right.$$
  (18) $$\sum_{k=1}^{n}A_{j}\left(s_{k}\right)=1.$$
5. Balanced assignment of the sensor-nodes to RPs as much as possible to prevent hot-spot phenomenon.
  ∀i=1,…,n and ∀j=1,…,n:
  (19) $$X_{i}\left(s_{j}\right)=\left\{\begin{array}{cc} 1 & \mathrm{if}\mathrm{sensor}\;j\;\mathrm{cannot}\mathrm{contact}\mathrm{with}\;\mathrm{sensor}\;i\;\mathrm{directly},\\ 0 & \mathrm{else}. \end{array}\right.$$
  (20) $$N(s_{i})=\mathrm{Number}\mathrm{of}\mathrm{connections}\mathrm{entered}\mathrm{to}\mathrm{sensor}\;i.$$
  *and due to the distributed nature of our approach, we assume that the following assumption in Equation (21) always happens.*
  ∀i=1,…,n,∀j=1,…,n,∀k=1,…,mandi≠j;
  (21) $$\sum_{j=1}^{n}X_{i}\left(s_{j}\right).A_{k}\left(s_{j}\right)\geq 2.$$
  *then the following constraints ensure balanced energy-consumption.*
  ∀i=1,…,n,∀j=1,…,n,i≠j,∀l=1,…,mand∀k=1,…,m;
  (22) $$X_{i}\left(s_{j}\right)\times A_{l}\left(s_{j}\right)=1,$$
  (23) $$N\left(s_{l}\right)\leq N\left(s_{k}\right)$$

### 4.3. Limitation of MILP-Based Approach

In fact, the problem that is modeled in this section can be divided to three parts as follows:

- Identifying a finite set of sojourn locations from a continuous trajectory. Thus, there should be a function or methodology to do this procedure.
  (24) $$\begin{array}{c} f:\Gamma \to \Psi \;\;\mathrm{where}\\ \Gamma \to \infty \\ \Psi \to m \end{array}$$
- Selecting a suitable set of RPs for each sojourn location as described in Expressions (4) and (5).
- And finally assigning the rest of sensor-nodes to the related RPs as illustrated in Expression (6).

Obviously, the general solution of our problem consists of solving three set assignment problems which the sets are related to each other. The set assignment is in the category of combinatorial optimization problem and its NP-hardness is proved in [49,50]. Apparently, for small-scale networks a MILP solver can obtain the optimal solution. By increasing the scale of the network, the MILP solver cannot be used to solve the problem efficiently. Therefore, it is essential to design a polynomial-time heuristic to achieve optimal or near-optimal solution.

In addition, there is another problem which is specific to most of the WSNs. The MILP formulation assumes that all of the information about the mobile-sink and the sensor-nodes are known. Therefore, the set assignment problem can be treated by focusing on finding a solution with all of the known parameters. However, practically, the mobile-sink is not familiar with all of the sensor-nodes. And the sensor-nodes do not know the general distribution map in the network. Thus, our problem becomes more difficult and our proposed heuristic should address this issue by being distributed and independent from complete information about sensor-nodes.

## 5. Proposed Heuristic

By considering the distributed nature of our designated EWSNs, we propose three heuristics for determining RPs and sojourn locations along with constructing routing trees in the network. The main objective in our design stage was concerning on distributed nature of the problem and inserting environmental and systematic parameters in the decision processes.

### 5.1. Stage I and II: Determining Sojourn Locations and RPs

Generally, the main idea of identifying the sojourn locations in the network by considering the objective function of minimizing DDT in Equation (7) is to consider the locations on the trajectory that the sensor-nodes have denser distribution. Since at the beginning of network’s operation, the mobile-sink is not familiar with the sensor-nodes which are being distributed in the periphery of trajectory, it will have a tour to introduce itself to the sensor-nodes which are in the communication range of the mobile-sink. To recognize the dense areas for selecting sojourn locations and RPs, one of the simple solutions is to deliver this work to the mobile-sink. However, there are two main disadvantages for this solution. The first one is related to computational resources for the mobile sink and data harvesting time in the start-tour. In this case, at first the mobile-sink needs to complete the first tour to harvest all of the related information about sensor-nodes. It should move with a very slow average speed to send its information to the sensor-nodes and wait until receiving the reply messages before visiting other sensor-nodes. Obviously, in large-scale EWSNs this process will be very time consuming. And after collecting all of the information about the sensor-nodes, a central station or the mobile-sink should do the computation for determining the sojourn locations and RPs. The second disadvantage is related to the sensor-nodes which should communicate with the mobile-sink in case of receiving introduction message that is energy-consuming process since the reply message should provide adequate information for the process of selection.

To eliminate the mentioned limitations, we attempt to design a distribute heuristic that allocates the responsibility of selecting RPs to the sensor-nodes and the sojourn locations will be identifies based on the position of selected RPs. The following steps illustrate our overall approach in this stage and more details will be presented afterwards.

1. The mobile-sink moves in the trajectory at the first tour for introducing itself to the periphery sensor-nodes and when a sensor-node receives introduction message, it will consider itself as the candidate RP. The mobile-sink moves in the trajectory and broadcasts the introduction message. Therefore, in this stage the mobile-sink does not wait for reply message and by moving in the trajectory without stopping can accomplish this task.
2. The candidate RP starts to do the process of identifying its potentiality for becoming RP based on our designed Fuzzy Decision System (FDS) which is being described with details in Section 5.1.1.
3. The voting procedure will be started after the previous step to choose the most suitable sensor-nodes as RPs.
4. The mobile-sink determines its sojourn locations based on the selected RPs.
5. The rest of sensor-nodes will be assigned to the appropriate RPs based on our designed methodology for constructing balanced routing trees as explained in Section 5.2.
6. After identifying the sojourn locations, the mobile-sink stops in each sojourn location to harvest data from the RPs which are being assigned to that specific sojourn location. Since the sensor-nodes are event-driven and their data includes information about an (some) event(s), the mobile-sink should harvest all of the buffered data from the RPs. To determine the waiting-time, the mobile-sink broadcasts a message by arriving to a sojourn location to inform the RPs about its arrival. Then RPs reply a message which includes the amount of their buffered data. And finally based on the channel capacity, the mobile-sink can calculate the required waiting-time. It should be mentioned that if during the waiting-time a RP receives new data, it informs the mobile-sink to extend the waiting-time.

#### 5.1.1. Fuzzy Decision System

Since its first introduction in 1960 [51,52], Fuzzy Logic theory could gain a hug attention in various applications. The main objective in Fuzzy Logic theory is to overcome uncertainties in the environment by constructing a system that its parameters are understandable for humans.

**Figure 4:**
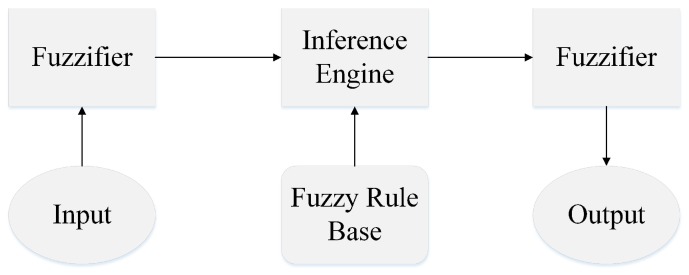
Fuzzy Inference System for identifying potentiality of the sensor-nodes to become RP.

Figure 4 depicts the general scheme of the Fuzzy Inference System that decides about the potentiality of a sensor-node to work as RP by taking input variables and calculating the output variable. The main advantages of this system are simplicity to implement in the sensor-nodes and robustness to deal with uncertainties in the environment. In our research, we have to determine the input and the output variables and design the Fuzzy rule base.

***Input Variables:*** Determining a suitable set of input variables for the Fuzzy Inference system has direct influence on the system’s accuracy. In the following, we will explain our input variables with details.

**Figure 5:**
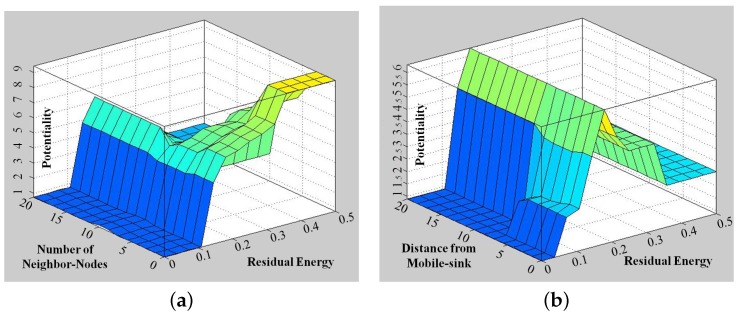
Fuzzy set for (**a**) Residual Energy and Number of Neighbors with Potentiality; (**b**) Residual Energy and Distance with Potentiality.

- *Residual energy* is the main factor for a sensor-node to be selected as RP. We designed the following linguistic variables to describe the amount of remaining energy for a sensor-node; *very high*, *high*, *medium*, *low*, and *very low*. Figure 5a illustrates the membership functions to map the crisp values of residual energy to the related linguistic values. The membership functions for the linguistic variables *high*, and *very low* are trapezoidal and triangular for the rest of linguistic variables.
- *Number of neighbor-nodes* is another factor that determines the popularity of a sensor-node in case of being selected as RP. In this factor, we just consider the sensor-nodes that can contact with the candidate-node directly. Therefore, a sensor-node with highest number of neighbor-nodes is more desirable. We defined three linguistic variables to describe the number of neighbor-nodes which are *high*, *medium*, and *low*. In Figure 5a, the membership functions for *high* and *low* are trapezoidal and for *medium* triangular.
- *Distance from Mobile-Sink* is the third factor in our designated Fuzzy inference system that is for obtaining the required energy for the sensor-node to contact with the mobile-sink in case of being selected as RP. Clearly, when a sensor-node is closer to the sojourn location, it can conserve its residual energy and extend the lifetime of the network. In the Fuzzy inference system, as shown in Figure 5b there are three linguistic variables to describe the distance from mobile-sink; *high*, *medium*, and *low*. The membership functions for *high* and *low* are trapezoidal and for *medium* the membership function is triangular.

***Output Variable:*** The output variable for the Fuzzy Inference System is called *Potentiality* which determines the degree of ability for a sensor-node to become a RP. We specify four linguistic variables which are *high*, *medium*, *low*, and *very low*. As shown in Figure 5, the membership functions for the linguistic variables *high* and *very low* are trapezoidal and for the rest of linguistic variables are triangular.

We designed the membership functions for all of the linguistic variables experimentally. As illustrated in Figure 4, after defining the linguistic values and the related membership functions, the next step is about obtaining the Fuzzy Rule Base. Table 1 consists of 45 Fuzzy rules which are based on the heuristic Fuzzy rule generation with the principle of balancing energy consumption among the sensor-nodes which can be elected as RP during the network’s operation with assuring the connectivity of the sensor-nodes. And the final step is defuzzification. In this step, the system should generate a crisp value for the amount of potentiality and the center of area (COA) is the technique that we applied.

#### 5.1.2. RP-Selection

We designed a distributed process to select RPs in the network. The main feature of a RP is its ability to contact with the mobile-sink, directly. To this end, identifying all of the sensor-nodes that can contact with the mobile-sink and grouping them in a set that is called Candidate-RP $\zeta =\left\{\rho_{1},\ldots ,\rho_{k}\right\}$ such that k≤|S| is the first step in our approach. Then the next step will be selecting the most suitable RPs in the network and our solution which is described in Algorithm 1 does this task. In Algorithm 1, Lines 1–3 ask from all of the candidate-RPs to calculate their potentiality for becoming a RP based on our designed fuzzy decision system. Lines 4–8 require the candidate-RPs to send their potentiality-score to the neighbor sensor-nodes. In Lines 9–11, the sensor-nodes which can contact with a candidate-RP directly are allowed to vote for it to become RP or not. And Lines 12–16 describe the general rule that if more than half of the neighbor sensor-nodes vote for a candidate-RP, it can be selected as RP. However, Lines 17–26 consider one of the situation in which a sensor-node votes for a candidate-RP that could not be selected as RP. In addition, that sensor-node cannot contact with another RP. In this situation, the sensor-node will send a special message to the candidate-RP and it becomes a RP.

#### 5.1.3. Sojourn Locations Selection

Determining a set of sojourn locations for the mobile-sink is another problem that arises after selecting the RPs in the network by assuming a fixed trajectory. Based on the communication technologies for the sensor-nodes and the mobile-sink two assumptions are possible to define the sojourn locations. In the first assumption, the sojourn locations are some specific points in the trajectory and the mobile-sink should stop in each point to harvest data from the RPs. In addition, in the second assumption, the sojourn locations are located in a communication range in which the mobile sink harvests data from the RP(s). In addition, in a fixed trajectory another assumption that can influence the sojourn locations is the sink-node and its installation manner. If the sink-node is being installed on a transportation vehicle or on an animal such as the approaches in [30,39], then defining some specific points or communication ranges as sojourn locations is not possible due to unpredictable (or hard to predict) behavior of the carrier objects that is not the focus of our current work. And if the sink-node is being installed on a controllable vehicle such as a robot then it is practical to characterize the sojourn locations. And generally, designing the sojourn locations follows a main strategy in which reducing the number of sojourn locations can reduce delay-time in the network.

**Algorithm 1:** RP-Node Selection**Input**: i) Candidate RPs, $\zeta =\left\{\rho_{1},\ldots ,\rho_{k}\right\}$, k≤|S|; ii) Neighbor-Nodes, $\rho_{i}=\left\{\eta_{1},\ldots ,\eta_{l_{i}}\right\}$, ∀i=1,…,k and $l_{i}\leq \left|S\right|$;
**Output**: i) RP-Nodes, $\Delta =\left\{\delta_{1},\ldots ,\delta_{m}\right\}$, m≤|S|; ii) The first group of sensor-nodes which follow the RPs, $\delta_{i}=\left\{\eta_{i},\ldots ,\eta_{l_{i}}\right\}$;

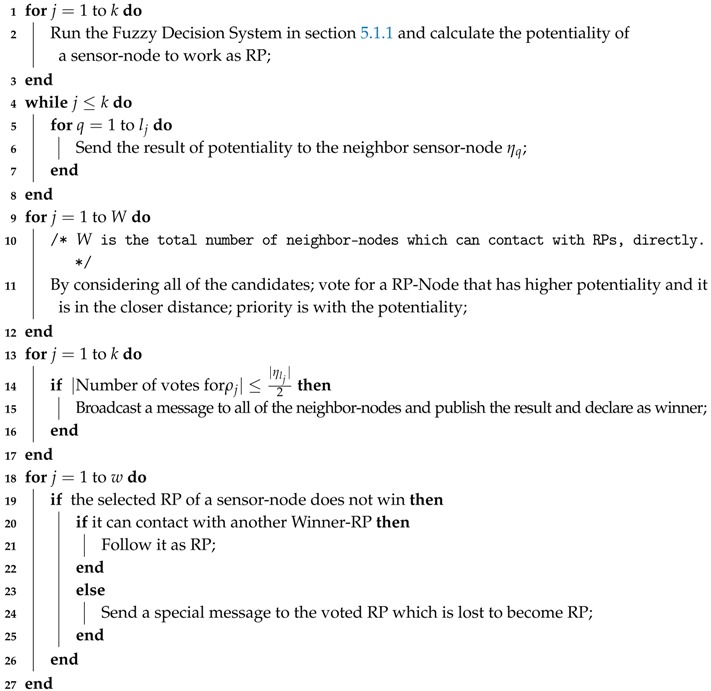


**Figure 6:**
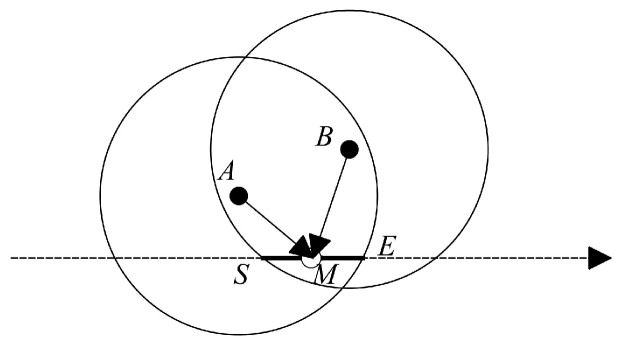
An example of two RPs in the communication range of each other which are located in the periphery of the trajectory.

In our research, we proposed a heuristic to select the sojourn locations after determining the RPs in the network. Since in our approach, it is assumed that the sojourn locations are some specific points that the mobile-sink stops to harvest data from the RPs, there will be three situations as follows:

1. If there is only one RP in the periphery of the trajectory and this RP cannot contact with other RPs, then the mobile-sink considers the closest distance to the RP as sojourn location.
2. If there are two RPs which are in the communication range of each other, as shown in Figure 6, we should balance energy consumption among the RPs for data communication. To illustrate our solution, we follow the example in Figure 6 in which there are two RPs *A* and *B*. The line $\bar{SE}$ specifies the intersected trajectory that the sojourn location should be selected for covering the two RPs. To obtain the position that can balance energy consumption, we select the middle of $\bar{SE}$ which is identified as *M*. By comparing $\vec{AM}$ with $\vec{BM}$ the point *M* moves to the direction that has larger amount of energy consumption in the line $\bar{SE}$. If $\vec{AM}=\vec{BM}$ then we can obtain the optimal solution. And if in $\bar{SE}$ we cannot obtain the optimal solution but obviously we can achieve to the most possible near optimal solution.
3. For the condition that there are more than two RPs in the periphery of the trajectory, we proposed Algorithm 2 that is described with details in the following.

**Figure 7:**
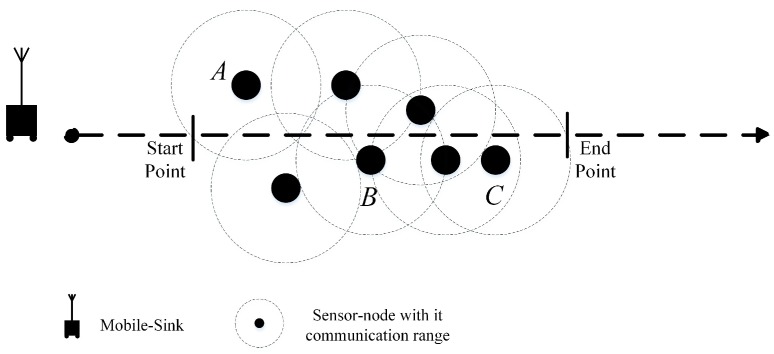
A communication range with more than two RPs in which the sojourn location(s) should be selected.

Figure 7 illustrates the situation in that there are more than two RPs in the periphery of the trajectory. Since the main task in the process of selecting the sojourn locations is to minimize the delay-time, in Algorithm 2, we concentrate on choosing a point that can cover all of the RPs which are in the communication range of each other. However, as shown in Figure 7, by selecting a sojourn location which can cover the RP *C*, the RP *A* and/or the RP *B* cannot send data to that sojourn location. Therefore, it is essential to select at least one more sojourn location in that specific part of the trajectory.

In Algorithm 2, we designed an effective method to select a set of appropriate sojourn locations. The input of the algorithm is a set of RPs $\Delta =\left\{\delta_{1},\ldots ,\delta_{m}\right\}$ such that m≤|S| and a set of sub-trajectories $\Gamma =\left\{\gamma_{1},\gamma_{2},\ldots ,\gamma_{l}\right\}$ such that l≤|Δ| and each sub-trajectory is in the communication range of at least one RP (The mobile-sink can harvest all of the related information about RPs and sub-trajectories by completing one tour and then runs our algorithm to identify the set of sojourn locations). Lines 2–4 consider the condition in which there is only one RP in relation with a sub-trajectory. And Lines 5–13 address the situation in that there are only two RPs in corresponding with a sub-trajectory. And finally Lines 14–22 discusses about the existence of more than two RPs for a sub-trajectory. In Line 15 the function $MaxPoint(\gamma_{i})$ finds the point(s) in the sub-trajectory $\gamma_{i}$ that if the mobile-sink stands in that point(s), it can cover the maximum number of RPs. The function $MaxLine(\gamma_{i})$ in Line 16 does the same process but it is for the situation to find a continues line in the sub-trajectory that covers the maximum number of RPs. Line 17 converts the continuous line from the previous step to one specific point with the condition for finding a point that minimizes total energy consumption for RPs to do data transformation and in case of equal values it considers the middle point. Between all of the candidate points in Line 18 the function MidPoint(Υ) finds the middle point or the point which is closer to the real middle point in the sub-trajectory among the candidate sojourn points. Afterward, that point will be selected as the first sojourn location in the sub-trajectory. In Line 20 the function score() determines the score of other candidate sojourn locations based on the current selected sojourn location. The procedure of scoring is to consider the current sojourn location and the candidate sojourn locations. Then the function for each candidate sojourn location identifies the number of non-intersected RPs with the candidate sojourn location. And finally if the score for other candidate sojourn locations becomes “zero”; it means the selected sojourn locations in the sub-trajectory can cover all of the RPs.

**Algorithm 2:** Determining Sojourn Locations**Input**: i) RP-Nodes, $\Delta =\left\{\delta_{1},\ldots ,\delta_{m}\right\}$, m≤|S|; ii) The trajectory of the mobile-sink, $\Gamma =\left\{\gamma_{1},\gamma_{2},\ldots ,\gamma_{l}\right\}$, l≤|Δ|;
**Output**: i) Sojourn locations, $\Psi =\left\{\psi_{1},\ldots ,\psi_{k}\right\}$, k≤m;

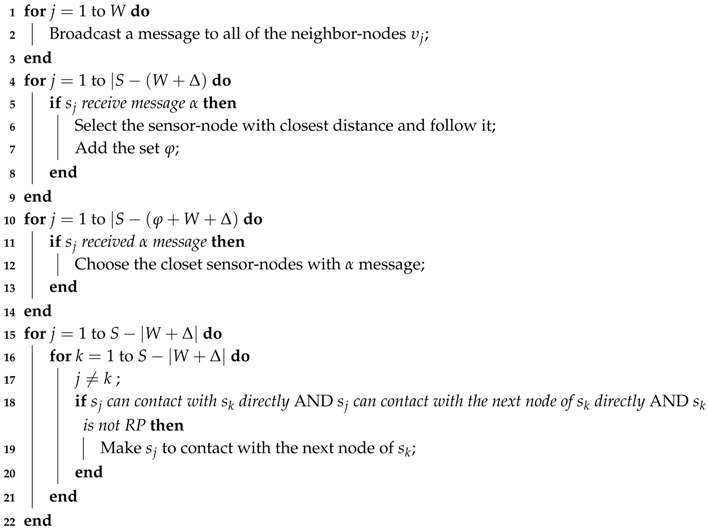


### 5.2. Stage III: Constructing the Routing Tree

The final stage of our proposed heuristic is about constructing the routing tree for each RP. Our emphasize in this process is constructing a balanced routing tree in which the sensor-nodes can contact with the RP with less amount of hops. As explained in Algorithm 3 the sensor-nodes which participate in the voting process start to broadcast a special message called α to the rest of sensor-nodes, in Line 2. When a sensor-node receives the α message(s) from other sensor-nodes, it follows the closest sensor-node. When all of the sensor-nodes could reach to their related RP, from Lines 15–22 a process begins that identifies triangles between sensor-nodes as illustrated in Section 4.2.2 in the Constrain (5) and removes the triangles in the network.

**Algorithm 3:** Constructing Balanced Routing Tree**Input**: i) The set of all the sensor-nodes that can contact with RPs, directly, $\Upsilon =\left\{\upsilon_{1},\ldots ,\upsilon_{w}\right\}$; **Output**: i) Routing Tree for each RP; 
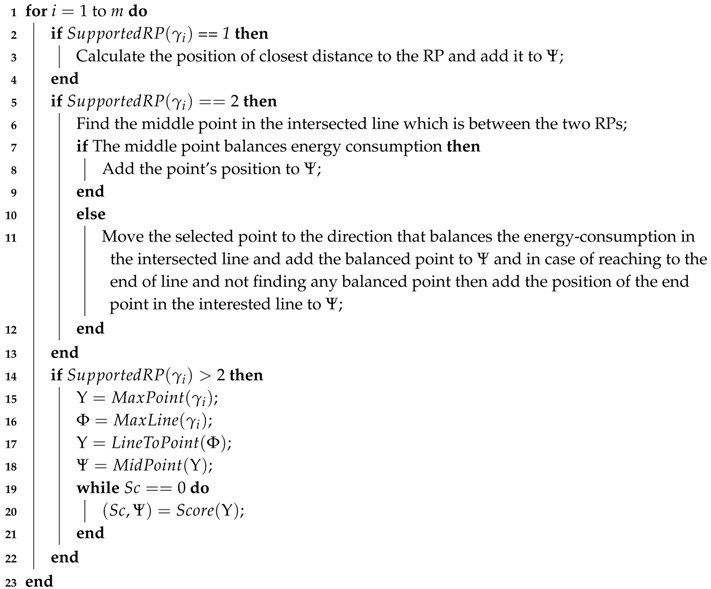


## 6. Numerical Results

In the current section, we illustrate the effectiveness of our proposed approach by providing numerical experiments. As discussed in the previous sections, we assume a fixed (arbitrary) trajectory in the environment. Therefore, in this section we compare our approach (FRM) with the approach in [39] (CBS) since it considers a fixed trajectory such as roaming a (some) public bus(es) in the city.

### 6.1. Parameter Settings

To simulate our experiments, we used MATLAB R2014a (with the platform Intel CPU Core-i7-3770, 16GB RAM, and Windows 7 operating system) as our tool that is popular in the research community related to WSNs and application of mobile-sink(s) [53]. We designed four experimental scenarios which are called WSN#1, WSN#2, WSN#3, and WSN#4 to verify our proposed approach as illustrated in Table 2. The main characteristics of our designed scenarios are the number of sensor-nodes and the size of development area. In addition, WSN#1 and WSN#2 are different based on their aggregation rates and same consideration for WSN#3 and WSN#4. Also, we adopt the concept of "round" in our experiments that indicates the period of times for changing the duty of the sensor-nodes in case of necessity. Each round consists of five tours for the mobile-sink.

### 6.2. Network Life-Cycle

**Figure 8:**
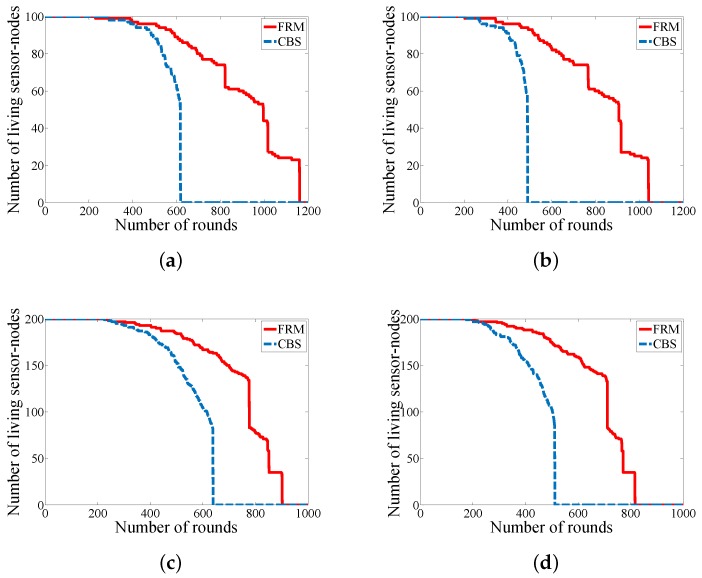
Comparison in case of network life-cycle (**a**) WSN#1; (**b**) WSN#2; (**c**) WSN#3; and (**d**) WSN#4.

Network life-cycle is an important parameter in the area of WSNs to identify the robustness of a proposed approach by studying the number of alive sensor-nodes during the network’s operation. Figure 8 depicts the comparison of FRM with CBS in case of network life-cycle that proves, generally, our approach can last longer and it balances the energy consumption among the sensor-nodes which protects the network from a dramatic death.

The main reason for the phenomenon of dramatic death in case of assuming a fixed trajectory in the environment is related to the hot-spot phenomenon. In fact, the sensor-nodes which are in the periphery of the trajectory due to their duties in the network consume a higher amount of energy in contrast with the sensor-nodes which are located far away from the trajectory. Therefore, in this scenario it is important to design an approach to avoid this phenomenon. And our proposed approach can reach to this goal by defining layers of duties from the sensor-nodes and preventing from unnecessary communication among the sensor-nodes.

### 6.3. Energy Consumption

**Figure 9:**
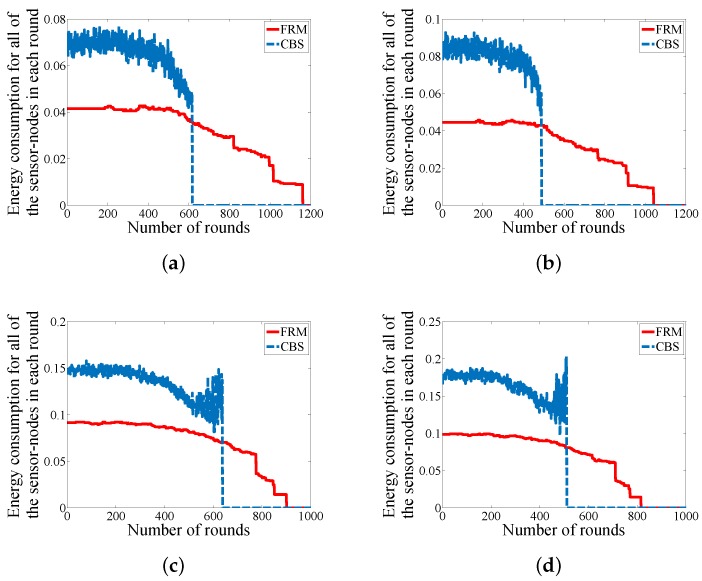
Comparison in case of energy consumption for all the sensor-nodes in each round (**a**) WSN#1; (**b**) WSN#2; (**c**) WSN#3; and (**d**) WSN#4.

Due to the battery-driven nature of WSNs (whole network or some parts of the network), it is essential to study the amount of energy consumption to validate a proposed approach. The key factor in energy consumption is to reduce data communication among the sensor-nodes. Figure 9 declares the amount of energy consumption for all of the sensor-nodes in each round. By analyzing the four development scenarios in the figure, there are three achievements for FRM in contrast with CBS. We could balance energy consumption among the sensor-nodes in each round as the smooth line for energy consumption in each round indicates. The second achievement is reducing the total amount of energy consumption for each round because our approach eliminates all of the unnecessary hierarchical structures and attempts to reduce the number of hops in case of multi-hop communication for the sensor-nodes. And the third achievement of our proposed approach is extending the operation time of the network.

**Figure 10:**
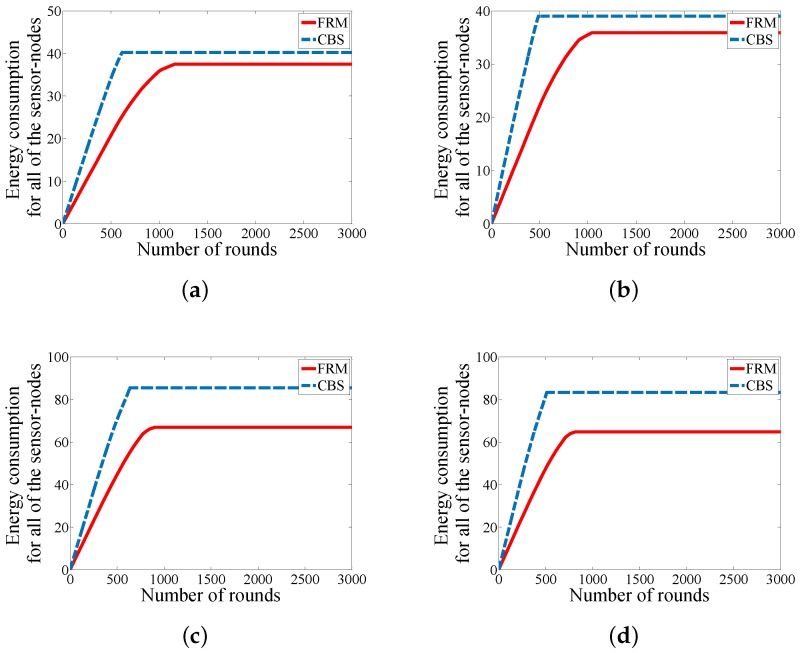
Comparison in case of energy consumption for all of the sensor-nodes (**a)** WSN#1; (**b**) WSN#2; (**c**) WSN#3; and (**d**) WSN#4.

Finally, Figure 10 presents the amount energy consumption for the whole network by increasing the number of rounds. Clearly our approach can conserve a considerable amount of energy in the network which can be used to reduce the size of batteries in the sensor-nodes for the applications in which the size of the sensor-nodes is an important factor such as battle field monitoring for hiding the sensor-nodes from the adversary.

## 7. Conclusions

In this paper, we study the problem of employing a mobile-sink into the WSNs by emphasizing on event-driven sensor-nodes. Due to the limitation of grounded robots or vehicles in the environment that should travel on the smooth roads and possible obstacles which restricts the freedom of the mobile-sink in path-selection, we focused on designing a framework for the sensor-nodes in the network to transfer the captured data to the mobile-sink which moves in a fixed trajectory. We formulated the problem by a Mixed Integer Linear Programming (MILP) problem to identify its constrains and expectations. In addition, we also proved the NP-hard nature of the problem that could lead us to propose three heuristics to achieve a comprehensive framework. The first heuristic finds a suitable set of RPs in the periphery of the trajectory. In this step, we designed a Fuzzy Decision System (FDS) which is a powerful tool to deal with uncertainties in the environment. Afterwards, in the second heuristic we proposed our solution to identify a suitable set of sojourn locations for the mobile-sink to harvest data from the RPs. And our third heuristic considers the problem of constructing routing trees for the sensor-nodes for sending their data to the related RPs. In the experimental section, we prove the effectiveness of our framework by numerical simulations. Generally, our approach can extend the lifetime of the network. It balances energy consumption between all of the sensor-nodes. In addition, it protects the network from the phenomenon of dramatic death in which still most of the sensor-nodes have an adequate amount of energy.

## Figures and Tables

**Table 1:** Fuzzy Mapping Rules for Obtaining Potentiality.

*Residual Energy*	*Number of Neighbor-Nodes*	*Distance from Mobile-Sink*	*Potentiality*
Very High	High	High	Low
Very High	High	Medium	Low
Very High	High	Low	Low
Very High	Medium	High	Medium
Very High	Medium	Medium	Medium
Very High	Medium	Low	High
Very High	Low	High	High
Very High	Low	Medium	High
Very High	Low	Low	High
High	High	High	Low
High	High	Medium	Low
High	High	Low	Low
High	Medium	High	Medium
High	Medium	Medium	Medium
High	Medium	Low	High
High	Low	High	High
High	Low	Medium	High
High	Low	Low	High
Medium	High	High	Medium
Medium	High	Medium	Medium
Medium	High	Low	Medium
Medium	Medium	High	Low
Medium	Medium	Medium	Medium
Medium	Medium	Low	High
Medium	Low	High	Medium
Medium	Low	Medium	Medium
Medium	Low	Low	Medium
Low	High	High	Very Low
Low	High	Medium	Very Low
Low	High	Low	Low
Low	Medium	High	Very Low
Low	Medium	Medium	Very Low
Low	Medium	Low	Low
Low	Low	High	Very Low
Low	Low	Medium	Very Low
Low	Low	Low	Low
Very Low	High	High	Very Low
Very Low	High	Medium	Very Low
Very Low	High	Low	Very Low
Very Low	Medium	High	Very Low
Very Low	Medium	Medium	Very Low
Very Low	Medium	Low	Very Low
Very Low	Low	High	Very Low
Very Low	Low	Medium	Very Low
Very Low	Low	Low	Very Low

**Table 2:** Experimental parameter settings [39,54].

Parameter	WSN#1	WSN#2	WSN#3	WSN#4
Area (L × L)	100 × 100 $m^{2}$	100 × 100 $m^{2}$	200 × 200 $m^{2}$	200 × 200 $m^{2}$
Number of sensor-nodes	100	100	200	200
Initial energy of the sensor-nodes	0.5 J	0.5 J	0.5 J	0.5 J
Communication radius of sensor-nodes	10 m	10 m	20 m	20 m
$E_{elect}$	50 nJ/bit	50 nJ/bit	50 nJ/bit	50 nJ/bit
$\xi_{fs}$	10 pJ/bit/$m^{2}$	10 pJ/bit/$m^{2}$	10 pJ/bit/$m^{2}$	10 pJ/bit/$m^{2}$
$\xi_{mp}$	0.0013 pJ/bit/$m^{4}$	0.0013 pJ/bit/$m^{4}$	0.0013 pJ/bit/$m^{2}$	0.0013 pJ/bit/$m^{2}$
Energy for data aggregation ($E_{DA}$)	5 nJ/bit	5 nJ/bit	5 nJ/bit	5 nJ/bit
Aggregation rate	0.6	0.8	0.6	0.8
Control packet	200 bits	200 bits	200 bits	200 bits
Message packet	4000 bits	4000 bits	4000 bits	4000 bits
